# Shape Information Improves the Cross-Cohort Performance of Deep Learning-Based Segmentation of the Hippocampus

**DOI:** 10.3389/fnins.2020.00015

**Published:** 2020-01-24

**Authors:** Irene Brusini, Olof Lindberg, J-Sebastian Muehlboeck, Örjan Smedby, Eric Westman, Chunliang Wang

**Affiliations:** ^1^Division of Biomedical Imaging, Department of Biomedical Engineering and Health Systems, KTH Royal Institute of Technology, Stockholm, Sweden; ^2^Division of Clinical Geriatrics, Department of Neurobiology, Care Sciences and Society, Karolinska Institute, Solna, Sweden

**Keywords:** hippocampus, brain MRI, Alzheimer’s disease, image segmentation, deep learning, statistical shape model

## Abstract

Performing an accurate segmentation of the hippocampus from brain magnetic resonance images is a crucial task in neuroimaging research, since its structural integrity is strongly related to several neurodegenerative disorders, including Alzheimer’s disease (AD). Some automatic segmentation tools are already being used, but, in recent years, new deep learning (DL)-based methods have been proven to be much more accurate in various medical image segmentation tasks. In this work, we propose a DL-based hippocampus segmentation framework that embeds statistical shape of the hippocampus as context information into the deep neural network (DNN). The inclusion of shape information is achieved with three main steps: (1) a U-Net-based segmentation, (2) a shape model estimation, and (3) a second U-Net-based segmentation which uses both the original input data and the fitted shape model. The trained DL architectures were tested on image data of three diagnostic groups [AD patients, subjects with mild cognitive impairment (MCI) and controls] from two cohorts (ADNI and AddNeuroMed). Both intra-cohort validation and cross-cohort validation were performed and compared with the conventional U-net architecture and some variations with other types of context information (i.e., autocontext and tissue-class context). Our results suggest that adding shape information can improve the segmentation accuracy in cross-cohort validation, i.e., when DNNs are trained on one cohort and applied to another. However, no significant benefit is observed in intra-cohort validation, i.e., training and testing DNNs on images from the same cohort. Moreover, compared to other types of context information, the use of shape context was shown to be the most successful in increasing the accuracy, while keeping the computational time in the order of a few minutes.

## Introduction

Alzheimer’s disease (AD) is a chronic progressive neurodegenerative disorder that constitutes approximately 60–70% of all dementia cases (Burns and Iliffe, 2009). The disease is characterized, since its first stages, by the loss of synapses and the depositions of certain lesions in several regions of the brain, which mainly include extracellular Aβ amyloid plaques and intracellular tau neurofibrillary tangles (Vinters, 2015). Moreover, on a macroscopic level, one of the most characteristic signs of the disease is brain atrophy, which is present in the majority of AD patients and can be estimated from magnetic resonance imaging (MRI) (Pini et al., 2016). Therefore, it is important to study imaging biomarkers that could allow early identification of subjects at risk of developing the disorder, as well as quantitatively reflect the disease’s level of progression. For example, such biomarkers should be able to distinguish AD both from the healthy state and from mild cognitive impairment (MCI). Indeed, MCI subjects constitute a relevant study group for the early identification of the disease, since several MCI cases, especially when presenting memory dysfunction, have a high probability of later evolving toward AD (Vinters, 2015).

According to the Braak criteria for AD staging (Braak and Braak, 1991), the progression of the disease starts from the transentorhinal cortex (stages I and II), involving then the hippocampus (stages III and IV), and finally spreading to the neocortex (stage V). These steps of progressions were defined based on the changes of accumulation of the neurofibrillary tangles. However, similar patterns can also be seen in the progression of brain atrophy according to multiple MRI studies, which have shown that the atrophy of the hippocampus measured from MRI images can be used, together with the atrophy of the entorhinal cortex, as an early sign of AD (Scheltens et al., 2002). By accurately measuring the volume of these two brain regions, it is possible to separate healthy subjects from AD patients with high precision (Liu et al., 2010). Moreover, shape analysis of the hippocampus has also been shown to be a valid tool for diagnosing AD and differentiating it from other forms of dementia (Lindberg et al., 2012). Evident patterns of hippocampal atrophy have also been reported in several neuroimaging studies on subjects with MCI (Tabatabaei-Jafari et al., 2015).

To properly assess the geometrical features (e.g., volume and shape) of the hippocampus, it is important to have accurate segmentation tools. Ideally, this should be done by completely automated software, since manual segmentation performed by an expert is both extremely time-consuming and relatively subjective. Various software that performs automatic hippocampal segmentation—as well as other brain image processing operations—already exists and is being widely used, for example, in the case of FreeSurfer (Fischl, 2012) or FSL (Jenkinson et al., 2012). However, the computational time of these well-known softwares for performing segmentation is often not acceptable for use in the clinical routine. Moreover, reaching a good segmentation accuracy is a challenging task due to several factors, including, for example, variations in MRI scanners and acquisition modalities, image artifacts, or variations in the brain due to the presence of pathology (Akkus et al., 2017), e.g., hippocampal atrophy.

Several previous studies have explored alternative approaches for automatic brain parcellation. Some of the most popular and successful ways to segment brain MRI images into structures of interest are atlas- and multi-atlas-based segmentation, which consist of integrating information present in brain MRI atlases registered to the target image by using different possible label fusions methods (Cabezas et al., 2011; Asman and Landman, 2013; Wang and Yushkevich, 2013; Pipitone et al., 2014). On the other hand, relevant improvements in the field of medical image segmentation have also been obtained by applying other techniques, such as statistical shape models (Leventon et al., 2000) or the further integration of tissue classifications into multi-atlas-based segmentation (Heckemann et al., 2010). In recent years, very good results have been achieved also by using deep learning (DL)-based methods, which are being more and more widely used because of their superior performance in very diverse medical image segmentation tasks (Ronneberger et al., 2015; Shelhamer et al., 2017). Therefore, such methods—and, in particular, those based on the use of convolutional neural networks—have recently been employed also in several studies on hippocampal segmentation (segmented either alone or together with other brain structures) achieving promising results (Kim et al., 2013; Milletari et al., 2017; Chen et al., 2018; Thyreau et al., 2018).

To further improve the segmentation accuracy, it is general practice to incorporate some context information into the segmentation frameworks. The use of context information, which enables the inclusion of likelihood and priors into the segmentation pipeline, has played an important role in computer vision (Oliva and Torralba, 2007; Tu and Bai, 2010). One example of context information, which has been widely applied in medical image segmentation tasks, is the so-called autocontext. This approach consists of first training one classifier and subsequently using its output as input to a second classifier (Tu and Bai, 2010; Chen et al., 2016; Mirikharaji et al., 2018). Several recent studies have suggested that applying the same strategy to deep neural networks (DNNs) could also improve the segmentation accuracy of brain structures (Chen et al., 2018). Another type of context information can be the tissue-class (Heckemann et al., 2010). More recently, shape context was proposed to help artificial neural networks to segment brain structures (Mahbod et al., 2018). This approach was later extended to DNNs in recent studies that demonstrated how the inclusion of shape priors into the segmentation pipeline can increase the robustness of the network’s segmentation accuracy. Such priors were successfully employed, for example, by adding a convolutional autoencoder to a traditional U-Net as shape regularization network (Ravishankar et al., 2017), by feeding a statistical shape model as an additional input to a fully convolutional network (FCN) (Wang and Smedby, 2017), by implementing a Bayesian model that incorporates a shape prior into a DL-based segmentation result (Ma et al., 2018), as well as by jointly training an FCN with a level set (Tang et al., 2017).

In this paper, we investigate whether the integration of shape information can improve the accuracy also in the context of hippocampal segmentation. We analyze the robustness of the method by both testing it on three different diagnostic groups of interest [healthy controls (HCs), MCI subjects, and AD patients] and validating it on a different cohort than the one used for training. Moreover, we compare the effect of adding shape context with two other types of context information: auto-context and tissue-class context. The inclusion of shape information is obtained by building FCNs that receive as input both a T1-weighted MRI image and a statistical shape model of the hippocampus, as already proposed in a previous study on a different segmentation task (Wang and Smedby, 2017). This is done by limiting preprocessing as much as possible, in order to obtain a very fast segmentation (in the order of a few minutes) that could potentially be integrated in the clinical routine.

## Materials and Methods

### Dataset

For training the networks and validating their performance, 54 T1-weighted structural brain MRI images from the cohort of the Alzheimer’s Disease Neuroimaging Initiative (ADNI) (Jack et al., 2008) were used. The primary goal of ADNI has been to test whether serial MRI, positron emission tomography (PET), other biological markers, and clinical and neuropsychological assessment can be combined to measure the progression of MCI and early AD. All ADNI data are obtainable from the ADNI database^1^ and updated information is available at www.adni-info.org.

Each of the selected 54 images (of size 197 × 233 × 189, with a voxel size of 1 × 1 × 1 mm^3^) had already been manually labeled by experts according to the European AD Consortium and ADNI Harmonized Hippocampal Protocol (HarP) (Boccardi et al., 2015b). The used dataset includes images from scanners of different magnetic field strength (both 1.5 and 3 T) and from three different diagnostic groups: HCs, AD, and MCI. As shown in Table 1, the data were selected in such a way that every possible pair of magnetic field strength and diagnosis is represented by the same number of subjects (i.e., nine subjects). No further processing of the images was performed before using them for training the proposed DL pipelines.

**TABLE 1:** Description of the training and test datasets.

**Cohort**	**Magnetic field strength**	**HC**	**AD**	**MCI**	**Number of subjects**
**Training and validation of the network**					
ADNI	1.5 T	9	9	9	27
ADNI	3 T	9	9	9	27
*Total number of subjects used for training and validation:*					54
**Only testing**					
AddNeuroMed	1.5 T	15	9	13	37
ADNI	1.5 T	213	312	179	704
ADNI	3 T	1799	875	2570	5244
*Total number of subjects used only for testing:*					5985

*The table shows the frequency for the magnetic field strength of the scanners and the subjects’ diagnosis in the datasets used for training and testing the proposed hippocampal segmentation pipelines.*

Another dataset was then used for further testing the performance of the networks trained only on the above-described ADNI data. It consists of 37 subjects from the AddNeuroMed cohort (Lovestone et al., 2009; Simmons et al., 2009), including all the three analyzed diagnostic groups and acquired using scanners having a magnetic field strength of 1.5 T (see Table 1). All 37 MRI images are high-resolution T1-weighted volumes of size of 193 × 229 × 193, with voxel size 1 × 1 × 1 mm. This dataset was chosen because its acquisition protocols were designed in a way compatible with the one used for the ADNI cohort (Simmons et al., 2011; Westman et al., 2011a), so that it is possible to use those data with a DL network previously trained using ADNI data. However, differences in terms of MRI scanner types, image quality, and image size are inevitably present between the two datasets, so it is useful to test the networks on the AddNeuroMed data to check also their performance on images from a new unknown cohort. Moreover, for those 37 MRI images, manual hippocampal segmentations were performed by an expert by following the HarP protocol, so a ground-truth segmentation mask was available.

Finally, the trained networks were tested also on a separate large dataset from the ADNI cohort including 5948 T1-weighted brain images (see Table 1). For these data, ground-truth manual segmentation masks of the hippocampus were not available. However, segmentations from FreeSurfer 6.0—processed through TheHiveDB neuroimaging database (Muehlboeck et al., 2013)—could be employed to check their consistency with the result obtained from the DL pipeline.

### Segmentation Pipeline

**FIGURE 1:**
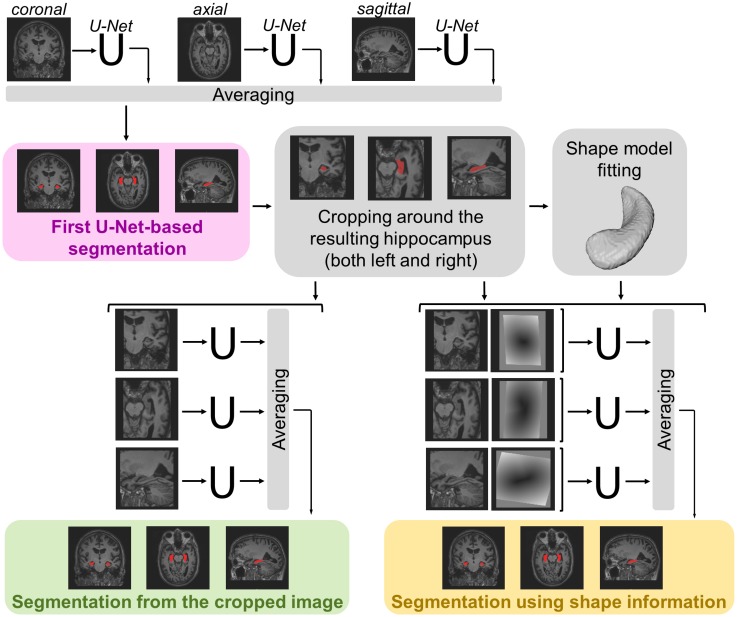
Schematic representation of the implemented segmentation pipelines. Hippocampal segmentation masks are created by applying all the three proposed hippocampal segmentation methods: MRI U-Net (in pink); Cropped MRI U-Net (in green); Shape MRI U-Net (in yellow). All networks receive T1-weighted MRI volumes as inputs, which, in the case of Cropped MRI U-Net and Shape MRI U-Net, are cropped around the hippocampus. Shape MRI U-Net also receives a second input channel that encodes *a priori* hippocampal shape information, which is represented as a distance map from the hippocampal surface (i.e., negative values inside the surface and positive values outside) obtained after a model fitting step.

The segmentation methods tested on the data described in the previous section consist of a maximum of three main steps (see Figure 1). For each method, a first 3D segmentation is performed using three orthogonal 2D U-Nets which take the original MRI image as input. This first segmentation approach is going to be referred to as *MRI U-Net*.

Moreover, a second segmentation method is presented, which adds a further step to the MRI U-Net. It consists of cropping the original MRI images around both the left and the right hippocampus (preliminarily segmented by using the MRI U-Net) and using the cropped images as input to other three orthogonal U-Nets. This approach is going to be referred to as *Cropped MRI U-Net.*

Finally, we propose a third approach that, after cropping the input MRI images, adds a further step consisting of fitting a statistical shape model to the segmentation obtained from MRI U-Net. Three other orthogonal U-Nets are employed, now taking two images as input: (1) the cropped MRI data and (2) their fitted shape model. This final methodology is going to be referred to as *Shape MRI U-Net*.

#### MRI U-Net

To perform the first DL-based segmentation, an FCN architecture was implemented: the so-called U-Net, proposed by Ronneberger et al. (2015), which has been shown to be particularly suitable for medical image segmentation tasks. One of its main strengths is that it can be applied to images of any size, providing as output a probabilistic label map whose dimension is proportional to that of the input image. This is achieved by replacing the fully connected layers of a classical convolutional neural network with more convolutional layers.

Since the segmentation needs to be performed on 3D brain volumes, we implemented three separate U-Nets, which make up the proposed *MRI U-Net* architecture. Each of these U-Net was trained independently to segment 2D slices acquired in one of the three orthogonal views (i.e., axial, coronal, or sagittal). The original T1-weighted image along with the manual segmentation is the only input given to the network for training. The final probability map of the hippocampal segmentation—including both the left and the right hippocampus—is generated by averaging the outputs of the three U-Nets. The final binary segmentation mask (pink box in Figure 1) is obtained by taking all voxels having a probability of belonging to either the left or the right hippocampus that is greater or equal to 0.5, i.e., which at least two of the U-Nets agree on classifying as hippocampus.

#### Cropped MRI U-Net

Once a binary segmentation mask has been obtained from MRI U-Net, it is possible to automatically discriminate the left from the right hippocampus by identifying the two major clusters of voxels in the segmentation mask using the tool “Cluster” from FMRIB Software Library (FSL) (Jenkinson et al., 2012). According to the orientation of the employed images, the right hippocampus is identified as the cluster whose center of gravity has the lowest *x* coordinate, while the left hippocampus is the remaining cluster.

Once the coordinates of the centers of gravity have been found, it is possible to crop the original MRI image around both the left and right hippocampus. In this way, from each subject, two new 3D volumes are obtained, each having the same predefined size (i.e., 87 × 105 × 111). These cropped volumes are used as input to the three new orthogonal U-Nets, making up the *Cropped MRI U-Net* architecture. Also in this case, the final label map (green box in Figure 1) is estimated by averaging the outputs of the three U-Nets and thresholding all voxels having a probability that is greater or equal to 0.5.

#### Generation of the Shape Model

The volumetric statistical shape models proposed by Leventon et al. (2000) were employed to add the shape context to the DL pipeline. The segmentations used to generate the statistical model were 12 manual labels for the left hippocampus and 12 for the right hippocampus. The total 24 segmentations were obtained from 12 images from the ADNI dataset of 54 subjects. The selection of these images was performed in such a way that all diagnostic groups were equally included (i.e., four HC, four AD, and four MCI), so that the model could represent the variability given by the different diagnoses without overfitting to a specific group. Moreover, all the selected images were acquired from scanners having a magnetic field strength of 3 T, in order to create the model from data of the highest quality available. The choice of the four subjects for each diagnostic group was performed randomly.

Three main modifications were performed on the manual labels. First, each of the 12 images was cropped twice—once around the left and once around the right hippocampus—so that each cropped image only included a region of size 87 × 105 × 111 around one of the regions of interest, similarly to what was done for the input images described in Section “Cropped MRI U-Net.” A main advantage of using cropped images also for creating the shape model is the reduction of computational time, since we are not interested in analyzing the rest of the 3D volume that does not include the hippocampus. Second, all labels from the right hippocampi were mirrored in order to match the orientation of the left hippocampus and to create a unique model for both sides together. Finally, each segmentation was up-sampled from 1 to 0.5 mm voxels to improve the resolution of the model. This was done to include more structural details and at the same time avoid large images by limiting the volumetric shape representation to the cropped region.

To generate the model, the mean signed distance function of each of the 24 manually segmented regions was computed, together with five main variations extracted via principal component analysis. Once the model is created, it is possible to fit it to each label map derived from the previous step (presented in section “MRI U-Net”) by solving a level set function, as described by Leventon et al. (2000). This fitting step generates a customized hippocampal shape that deviates from the mean shape by adding those variations. The shape fitting step could potentially correct for possible segmentation errors and irregularities present in the MRI U-Net output.

#### Shape MRI U-Net

The segmentation masks derived from MRI U-Net are cropped around the centers of gravity of both the left and the right hippocampus. Each of these two segmented sides can be associated to its own shape context as described in the previous section. Such shape context consists of a distance map from the hippocampal surface obtained after the model fitting step described in the previous section. In the distance map, the hippocampal surface corresponds to the zero level set, while all voxels inside the surface have negative intensity values and all voxels outside have positive values.

After this, both the cropped original MRI volumes (described in section “Cropped MRI U-Net”) and the distance maps from the fitted hippocampal surface are used as inputs to three new U-Nets, which constitute the *Shape MRI U-Net* pipeline. Also in this case, the three networks are trained independently from scratch in the three different orthogonal views. The final segmentation mask (yellow box in Figure 1) is estimated by averaging the outputs of the three U-Nets and thresholding the voxels having a probability that is greater or equal to 0.5.

### Implementation Details

To build the DL architecture, we used the Keras framework^2^. The implemented U-Nets are identical to those proposed in the original paper by Ronneberger et al. (2015). However, to adapt the images to all the down- and up-sampling steps of the original implementation, all the original brain MRI data (having size 197 × 233 × 189) are resized to 208 × 224 × 192 for the first segmentation step, i.e., MRI U-Net. Instead, for the second (Cropped MRI U-Net) and third (Shape MRI U-Net) approaches, the input data (87 × 105 × 111) are resized to 96 × 112 × 112.

Two different data normalization methods are applied to the input T1-weighted volumes and the shape context images. For the latter ones, the voxel intensity is divided by the standard deviation (computed from all subjects) while keeping the reference point of 0 that corresponds to the hippocampal surface. As for the MRI scans, their intensities are first normalized individually by mapping the lower 5% cutting point of each subject’s histogram to 0 and the upper 5% to 1. Afterward, the images are also normalized all together by subtracting the group mean from all subjects and dividing the intensities by the group standard deviation, so that the normalized images have zero mean and a standard deviation of 1.

During the training phase, data augmentation is also employed by generating mini-batches of data in real-time. The data generator randomly applies rotations (within a range of ± 10°), width shifts (range of ± 0.1 ⋅ total image width), height shifts (range of ± 0.1 ⋅ total image height), and zooming (original 100% zoom ± 20%).

We used the negative Dice score as the loss function to be minimized during the training phase. The number of epochs was always set to 60 for all the three U-Nets of the first pipeline that uses only the T1-weighted volumes as inputs. Instead, for Cropped MRI U-Net and Shape MRI U-Net, the number of epochs was reduced to 40 for all the U-Nets.

### Alternative Segmentation Methods

The same images used for training and testing our architecture had also been segmented using the last version (6.0) of FreeSurfer^3^, a software tool for image analysis that is freely available online. It is one of the most commonly used tools for automatic segmentation of the subcortical white matter and deep gray matter volumetric structures, including the hippocampus (Fischl et al., 2002, 2004). For this reason, it was chosen as a reference method for comparison of the performance of the pipeline proposed in our work.

To better investigate the contribution given by adding the shape-model-fitting step, we compared the performance of our proposed pipelines with two alternative types of context information too. They were integrated in the following two networks:

#### Tissue MRI U-Net

The hippocampus is a gray matter structure that borders with other tissue types in specific locations, so *a priori* tissue type classification could help the network to identify the boundaries of the hippocampus. Therefore, three main tissue type segmentations (i.e., gray matter, white matter, and cerebrospinal fluid) are used as context input to the network to be integrated to the cropped MRI image. Such segmentations could be obtained automatically from the original MRI image by using the FMRIB’s Automated Segmentation Tool (FAST) from FSL (Zhang et al., 2001; Jenkinson et al., 2012). The network thus has four input channels in total: the cropped MRI image (obtained in the same way as in Cropped MRI U-Net and Shape MRI U-Net), as well as the three tissue segmentations (cropped in the same location as the T1-weighted images).

#### Autocontext MRI U-Net

The autocontext strategy is used: the cropped segmentation derived from *MRI U-Net* is given as second input channel to the network. This technique is one of the most well-known types of context information (Tu and Bai, 2010; Chen et al., 2016; Mirikharaji et al., 2018) and we aimed at investigating its effect also on our application of interest.

### Method Evaluation

#### Single-Cohort Evaluation

The proposed methods were first evaluated using ninefold cross-validation on the first dataset of 54 subjects from the ADNI cohort, for which manual hippocampal segmentations were available and used during training. For each fold, 48 of the cases were used for training and the remaining six for testing, and the test set always included all the six possible combinations of magnetic field strength and diagnosis presented in Table 1. When the shape context was included in the pipeline, the shape model described in Section “Generation of the Shape Model” was always the same and not re-created for each fold.

The evaluation metrics used to analyze the accuracy were the Dice score, precision, recall, and Hausdorff distance. The Dice score (Dice, 1945) is an index that measures the degree of overlap between two segmentation masks with values between 0 (no overlap) and 1 (matching segmentations). When comparing a hippocampal segmentation result with its ground truth, we can define as “true positives” (TP) the number of voxels correctly classified as belonging to the hippocampus and “false positives” (FP) those wrongly classified as belonging to the hippocampus. On the other hand, voxels correctly classified as background are “true negatives” (TN) and those wrongly classified as background are “false negatives” (FN). Given these definitions, we could estimate the Dice score as $\frac{2\,T\,P}{2\,T\,P+F\,P+F\,N}$, the precision as $\frac{T\,P}{T\,P+F\,P}$, and the recall as $\frac{T\,P}{T\,P+F\,N}$. Finally, as regards the Hausdorff distance, since it is a metric that tends to be highly sensitive to the presence of outliers (Huttenlocher et al., 1993; Taha and Hanbury, 2015), we applied the quantile method proposed by Huttenlocher et al. (1993). This method consists of, first of all, computing the closest distance between every point of a segmentation mask and the ground-truth. After this, these computed distances are sorted (from the lowest to the highest) and, instead of simply identifying their maximum value (i.e., the classical Hausdorff distance), their *q*th quantile is reported. In particular, in this paper, the 95th percentile of the distances was computed for each subject.

#### Cross-Cohort Evaluation

Once the segmentation pipelines had been trained and validated as described above, they were tested on a new unseen dataset of 37 subjects from the AddNeuroMed cohort. Since ground-truth segmentation masks were available for this dataset, the same evaluation metrics described in the previous section were used. Moreover, the performance differences between the methods were analyzed by carrying out pairwise comparisons between the evaluation metrics obtained from all the tested DL-based pipelines. This was done by defining a mixed-effect analysis of variance model with the segmentation methods and the sides (i.e., left or right) as fixed effects, the subjects as random effect, and the resulting evaluation metrics as dependent variables. The statistical calculation was performed in Stata 13.1 (StataCorp, College Station, TX, United States).

Finally, the same networks were also trained on the AddNeuroMed dataset and tested on the ADNI dataset, which had previously been used as training set. Dice score, precision, recall, and Hausdorff distance were computed. This was done in order to further investigate the effect of training the pipelines on data from a certain cohort and testing them on a new unseen cohort.

#### Evaluation on a Larger ADNI Dataset

As a final test of the trained networks, the proposed DL-based segmentation methods, which were trained on the above-described ADNI dataset of 54 subjects, were also applied on a separate larger dataset of 5948 T1-weighted brain images still from the ADNI cohort. Given the large amount of data and the consequently long time needed to perform all the segmentations, we only tested two pipelines in this phase: one not including shape information—MRI U-Net—and one including such information—Shape MRI U-Net.

For this dataset, ground-truth manual segmentation masks of the hippocampus were not available, but segmentations from FreeSurfer 6.0 could be easily obtained. Therefore, they were employed to check their consistency with the result obtained from the DL pipelines. This choice was motivated by the fact that FreeSurfer is still among the most commonly used software for brain image analysis. Thus, a similarity between our results and those from FreeSurfer could allow us to the test the potential of our methods to possibly replace one tool that is already well-known and established. Such consistency was analyzed by computing the correlation between the FreeSurfer volumes and those obtained through each DL-based pipeline. Moreover, two additional shape similarity metrics (i.e., Dice score and Hausdorff distance) were also computed to evaluate the similarity between the results from the proposed pipelines and those from FreeSurfer.

In addition, given the large amount of data in this dataset, we investigated whether our segmentation results could reflect the volumetric changes in the hippocampus between the three diagnostic group of interest, i.e., AD, MCI, and HC. This was done by selecting all subjects whose diagnosis did not change within 2 years after the first MRI scan. Subsequently, we computed, for each subject, the hippocampal volume at baseline and divided it by the total intracranial volume (ICV), which is one of the outputs measurements (eTIV, estimated total ICV) given by FreeSurfer 6.0. This normalization is often used in literature in order to have a more reliable estimation of atrophy caused by neurodegeneration (Voevodskaya et al., 2014). This measurement was then multiplied by the average ICV for all the subjects of interest. A one-way ANOVA test was then employed to identify whether a statistically significant difference (*p* < 0.05) could be found between the groups. Moreover, the normalized volumes were also used to fit three binary logistic regression models (i.e., for AD vs. HC, AD vs. MCI, and MCI vs. HC) to investigate the possibility of predicting the diagnosis of one subjects from the above-described volumetric measurements. In particular, each model was generated to provide as output the probability of a subject to belong to a certain diagnostic group as a function of the hippocampal volume multiplied by the ratio between the ICV and the specific subject’s ICV. The prediction power of each binary model was analyzed by computing three evaluations metrics: area under the curve (AUC), sensitivity, and specificity.

Finally, in this dataset, 2704 of the scans were repeated twice on the same subject, with the same scanner and at the same time point (within the same week). This allowed us to perform a test–retest analysis to make sure that the implemented methods are reproducible and consistent between the two subsequent scans. Therefore, for each of the tested methods, we computed the concordance correlation coefficient (CCC) (Lin, 1989) between the hippocampal volumes from the two subsequent scans. This coefficient describes the agreement between two different measurements of the same variable. CCC varies between –1 and 1, and CCC = 1 indicates perfect reproducibility.

## Results

### Single-Cohort Evaluation

The performance of the three types of context-aware segmentation methods was evaluated on the first ADNI dataset of 54 subjects through ninefold cross-validation and compared between the preliminary segmentation step (MRI U-Net) and the additional steps using only cropped data (Cropped MRI U-Net) or cropped data together with shape context (Shape MRI U-Net), as shown in Table 2. No relevant differences were found between the three methods, which showed quite consistent results on both the left and the right hippocampus. Among all the tested DL-based methods, Tissue MRI U-Net showed the worst performance, having a slightly lower accuracy and higher Hausdorff distance in average compared to the other methods.

**TABLE 2:** Single-cohort evaluation.

**Region of interest**	**Segmentation method**	**Dice score**	**Precision**	**Recall**	**Hausdorff distance (in voxels)**
Left hippocampus	MRI U-Net	90.17 ± 1.44%	89.46 ± 2.20%	90.96 ± 2.29%	2.33 ± 0.55
	Cropped MRI U-Net	90.28 ± 1.30%	89.36 ± 2.20%	91.28 ± 1.85%	2.22 ± 0.53
	Shape MRI U-Net	90.01 ± 1.41%	88.55 ± 2.69%	91.60 ± 1.87%	2.35 ± 0.67
	Tissue MRI U-Net	88.79 ± 1.61%	86.79 ± 2.72%	91.01 ± 2.94%	2.39 ± 0.60
	Autocontext MRI U-Net	89.45 ± 1.46%	86.69 ± 2.77%	92.53 ± 2.75%	2.30 ± 0.57
	FreeSurfer 6.0	79.52 ± 3.14%	82.94 ± 5.01%	76.60 ± 3.94%	4.34 ± 1.08
Right hippocampus	MRI U-Net	90.12 ± 1.41%	89.59 ± 2.48%	90.77 ± 2.72%	2.39 ± 0.53
	Cropped MRI U-Net	90.26 ± 1.41%	89.29 ± 2.62%	91.35 ± 2.39%	2.47 ± 0.59
	Shape MRI U-Net	90.08 ± 1.67%	88.50 ± 3.39%	91.86 ± 2.34%	2.54 ± 0.79
	Tissue MRI U-Net	88.74 ± 1.50%	86.4 ± 2.84%	91.00 ± 3.16%	2.63 ± 0.70
	Autocontext MRI U-Net	89.63 ± 1.32%	87.25 ± 2.81%	92.30 ± 2.89%	2.39 ± 0.53
	FreeSurfer 6.0	80.21 ± 3.86%	83.63 ± 4.35%	77.31 ± 5.36%	4.50 ± 1.23

*The performance of the proposed methods (in terms of Dice score, precision, recall, and Hausdorff distance) was computed through ninefold cross validation and compared with that of FreeSurfer 6.0. All evaluation metrics are expressed as mean ± standard deviation.*

**FIGURE 2:**
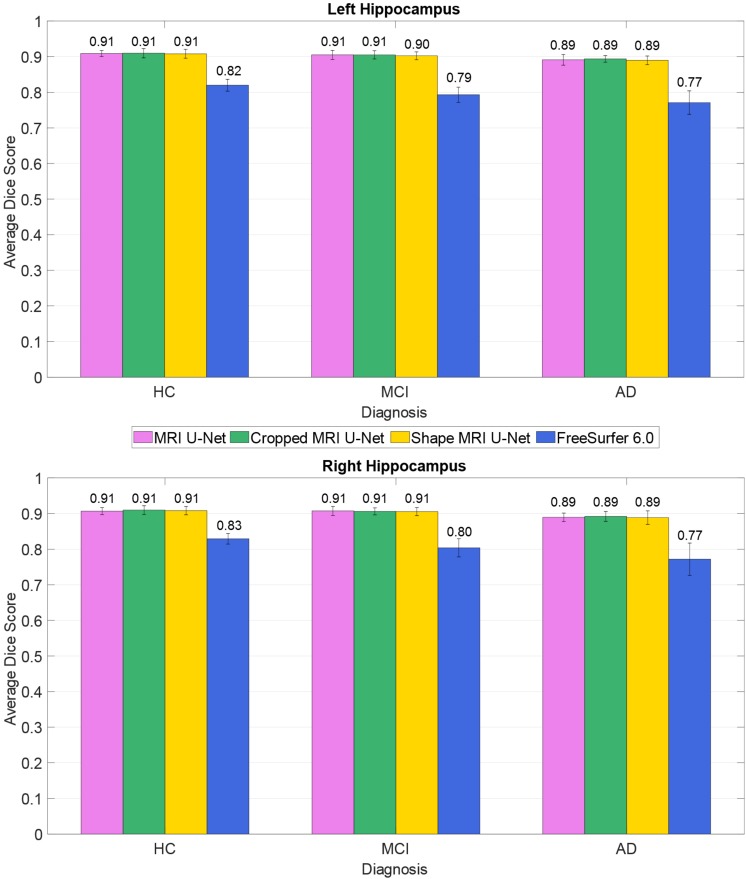
Difference in segmentation accuracy (from cross validation) between the three analyzed diagnostic groups (HC, MCI, and AD). The accuracy is expressed as the Dice score averaged across all subjects and is represented with histograms for each of the tested methods (see color legend). The error bars show the standard deviation of the Dice score.

The average Dice score was also estimated within each of the three diagnostic groups (HC, AD, and MCI). This was done to check whether the system has a consistent performance across all possible forms of hippocampal integrity. As shown in Figure 2, all diagnostic groups showed a similar segmentation accuracy in both the left and right hippocampus by using the three proposed methods. However, the AD patients always presented a slightly lower Dice score (1 or 2% lower in average) with respect to the other two subject groups.

As presented in Table 2, our methods yielded better values than FreeSurfer in all the considered evaluation metrics. This applies also for the comparison between diagnostic groups (see Figure 2), in which, contrary to the proposed DL methods, FreeSurfer showed a higher performance loss when dealing with MCI and—even more—AD subjects, compared to the HCs.

In order to better understand the influence of each of the three independent U-Nets (one for each view) toward the final segmentation, we also computed the evaluation metrics separately for each U-Net (see Supplementary Table S1). These results showed how, for MRI U-Net, the highest accuracy is obtained on the axial view. By contrast, for Cropped MRI U-Net and Shape MRI U-Net, the highest accuracy can be observed on the coronal view. However, while no big differences can be found across all views in MRI U-Net and Shape MRI U-Net, Cropped MRI U-Net showed an evident decrease in performance on the sagittal view in terms of Dice score, precision, and Hausdorff distance.

Moreover, the present methods were proven to be more efficient also in terms of computational time, at least when only a hippocampal segmentation is desired. On a personal computer with an Nvidia GTX 1080 graphic card and 32 GB of RAM, each segmentation took between 25 and 30 s with the simple MRI U-Net methodology. When performing one segmentation with Cropped MRI U-Net, approximately 1 min was taken. Using Shape MRI U-Net, about two and a half minutes was needed for one subject.

### Cross-Cohort Evaluation

#### Testing on the AddNeuroMed Dataset

When the proposed segmentation pipelines were tested on the new unseen dataset from the AddNeuroMed cohort, larger differences between the tested methods could be observed (see Table 3).

**TABLE 3:** First cross-cohort evaluation.

**Region of interest**	**Segmentation method**	**Dice score**	**Precision**	**Recall**	**Hausdorff distance (in voxels)**
Left hippocampus	MRI U-Net	79.09 ± 2.63%	74.72 ± 4.27%	84.23 ± 3.15%	3.44 ± 0.74
	Cropped MRI U-Net	84.44 ± 2.32%	78.47 ± 4.17%	91.60 ± 2.47%	3.19 ± 0.64
	Shape MRI U-Net	84.92 ± 2.56%	79.46 ± 5.03%	91.57 ± 3.60%	3.16 ± 0.77
	Tissue MRI U-Net	84.32 ± 2.16%	79.04 ± 4.12%	90.59 ± 2.90%	3.33 ± 0.85
	Autocontext MRI U-Net	80.55 ± 2.61%	73.99 ± 4.23%	88.67 ± 3.50%	3.33 ± 0.72
	FreeSurfer 6.0	79.41 ± 3.77%	78.89 ± 5.46%	80.20 ± 4.39%	4.24 ± 1.25
Right hippocampus	MRI U-Net	80.15 ± 2.25%	74.54 ± 3.12%	86.80 ± 3.08%	3.92 ± 1.14
	Cropped MRI U-Net	82.85 ± 2.52%	75.97 ± 3.91%	91.27 ± 2.31%	3.80 ± 1.05
	Shape MRI U-Net	84.19 ± 2.50%	77.94 ± 4.49%	91.83 ± 3.28%	3.62 ± 1.04
	Tissue MRI U-Net	82.88 ± 2.35%	76.86 ± 3.60%	90.08 ± 2.71%	3.68 ± 1.11
	Autocontext MRI U-Net	80.51 ± 2.20%	73.08 ± 3.27%	89.79 ± 3.03%	3.88 ± 1.16
	FreeSurfer 6.0	79.57 ± 3.54%	77.71 ± 5.53%	81.78 ± 3.40%	4.61 ± 1.11

*The performance of the proposed methods (in terms of Dice score, precision, recall, and Hausdorff distance) was tested on a new unseen dataset from a different cohort (i.e., AddNeuroMed cohort) than the one used for training. The performance is reported also for the segmentations obtained using FreeSurfer 6.0 on the same data. All evaluation metrics are expressed as mean ± standard deviation.*

The accuracy achieved by segmenting the MRI images using the MRI U-Net architecture is now very close to that obtained by using FreeSurfer 6.0. In particular, the two methods have almost identical Dice scores, while the precision and recall are, respectively, decreased and increased by using MRI U-Net. Moreover, FreeSurfer has a slightly higher Hausdorff distance in average.

When performing the segmentation using the other two proposed pipelines, an improvement in the performance can be observed. Dice score, precision, and recall positively increased by using the Cropped MRI U-Net architecture and, even more, the Shape MRI U-Net. The benefit of adding shape context was particularly noticed in the right hippocampus, where the average Dice score increased by 4.04% with respect to MRI U-Net (compared to + 2.70% obtained with Cropped MRI U-Net), the average precision by 3.40% (compared to + 1.43%), and the average recall by 5.03% (compared to + 4.47%). For the left hippocampus, the difference between Cropped MRI U-Net and Shape MRI U-Net was less evident, but in both cases, all evaluation metrics increased by approximately 4% with respect to MRI U-Net.

Also for this analysis, we calculated the evaluation metrics separately for each independent 2D U-Net (see Supplementary Table S2). Similarly to what has been obtained for the single-cohort analysis, MRI U-Net showed its best accuracy on the axial input slices, while with Cropped MRI U-Net and Shape MRI U-Net no relevant differences could be noticed between coronal and axial views. Moreover, most of the single 2D U-Nets of Cropped MRI U-Net showed a lower performance compared to Shape MRI U-Net, whose results are also more consistent across views. In particular, the sagittal 2D U-Net of Cropped MRI U-Net was still shown to have a very high Hausdorff distance compared to all other views and approaches, as well as particularly low Dice score and precision.

The two alternative integrations of context information did not achieve a better performance than the proposed methods. In particular, Autocontext MRI U-Net showed a very similar result to MRI U-Net. Instead, with Tissue MRI U-Net, the performance is comparable to that of Cropped MRI U-Net and Shape MRI U-Net, but never outperforming them in any of the analyzed evaluation metrics.

**FIGURE 3:**
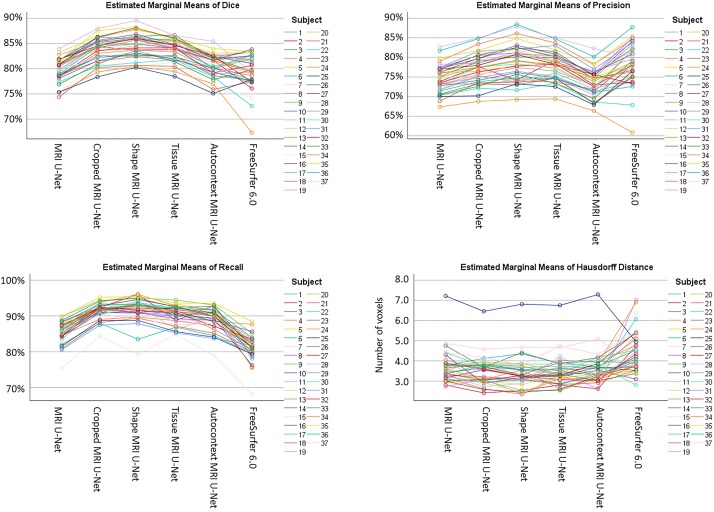
Variability of the performance within subjects on the AddNeuroMed test dataset. The plots are showing how, for each subject (see color legend), the evaluation metrics (on the vertical axes) change according to the segmentation method (on the horizontal axes) being used. Each of the four plots represents one specific evaluation metric: Dice score (top left), precision (top right), recall (bottom left), and Hausdorff distance (bottom right).

As shown in Supplementary Table S3, a statistically significant difference (i.e., *p* < 0.05 with Bonferroni correction) was found in the majority of the pairwise comparisons between the tested segmentation methods for all the evaluation metrics, except for the Hausdorff distance. The value of this latter metric is indeed quite consistent across all DL-based methods (except for the difference between Shape MRI U-Net and MRI U-Net, which resulted to be significant). Significantly larger Hausdorff distances were, however, always found when using FreeSurfer 6.0 as opposed to the pipelines implemented in the present work. Moreover, the choice of the subject to be segmented—and, subsequently, the image quality, as well as the level of degeneration—was found to highly influence the performance. Figure 3 shows how, for each subject, the evaluation metrics tended to vary with a consistent pattern according to the method being used and maintained a rather similar between-subject variability within each method.

**FIGURE 4:**
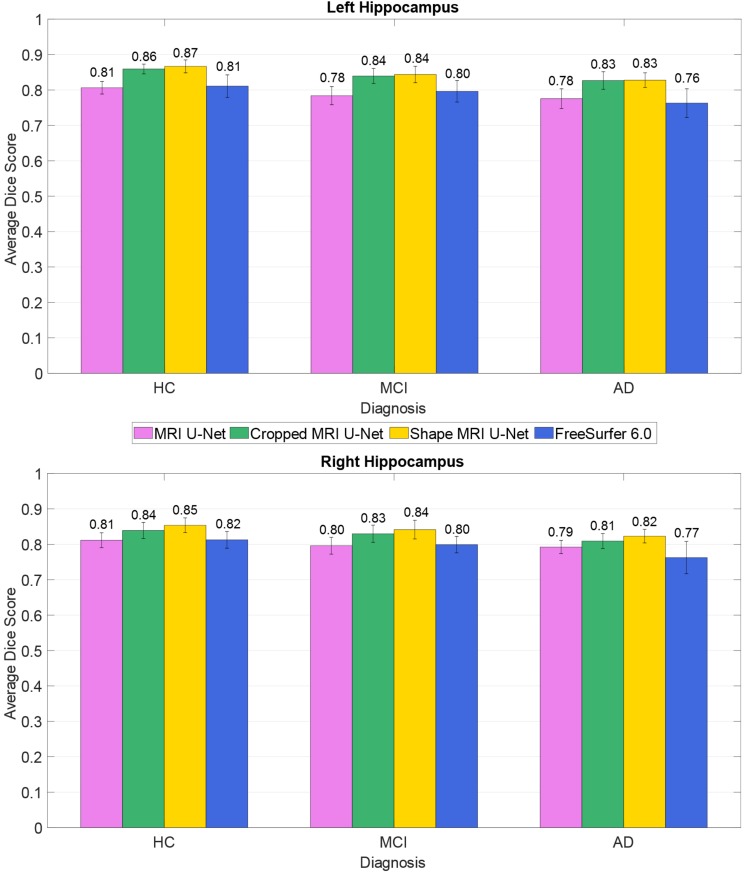
Difference in segmentation accuracy (on the AddNeuroMed test dataset) between the three analyzed diagnostic groups (HC, MCI, and AD). The accuracy is expressed as the Dice score averaged across all subjects and is represented with histograms for each of the tested methods (see color legend). The error bars show the standard deviation of the Dice score.

The average Dice scores for each diagnostic group from the AddNeuroMed dataset were also analyzed, as shown in Figure 4. The results reflect what has been observed on the whole dataset (i.e., averaging the results across all 37 subjects): both Cropped MRI U-Net and Shape MRI U-Net showed a superior accuracy compared to MRI U-Net, and in general the DL-based methods performed better than FreeSurfer 6.0.

#### Testing on the ADNI Dataset

All the implemented networks were re-trained using the data from the AddNeuroMed cohort in order to be tested on the 54 subjects from the ADNI cohort that had previously been used for training. Average Dice score, precision, recall, and Hausdorff distance were computed and presented in Table 4. The results are rather consistent with what has been found for the first cross-cohort evaluation presented in Section “Testing on the AddNeuroMed Dataset.” The average Dice scores for MRI U-Net and Autocontext MRI U-Net are very similar to those obtained using FreeSurfer 6.0, while they increase when using Cropped MRI U-Net and Shape MRI U-Net. However, in this case, the best results in terms of Hausdorff distance could be found in Autocontext MRI U-Net, followed by the Shape MRI U-Net implementation.

**TABLE 4:** Second cross-cohort evaluation.

**Region of interest**	**Segmentation method**	**Dice score**	**Precision**	**Recall**	**Hausdorff distance (in voxels)**
Left hippocampus	MRI U-Net	80.26 ± 3.93%	87.92 ± 3.72%	74.03 ± 5.48%	3.52 ± 0.85
	Cropped MRI U-Net	84.56 ± 2.45%	88.12 ± 2.77%	81.42 ± 4.00%	3.44 ± 0.82
	Shape MRI U-Net	85.06 ± 2.47%	87.85 ± 3.08%	82.57 ± 3.72%	3.34 ± 0.74
	Tissue MRI U-Net	73.39 ± 8.93%	75.66 ± 8.90%	71.40 ± 9.45%	4.30 ± 1.04
	Autocontext MRI U-Net	79.64 ± 7.50%	77.37 ± 7.84%	82.14 ± 7.56%	3.00 ± 0.75
	FreeSurfer 6.0	79.52 ± 3.14%	82.94 ± 5.01%	76.60 ± 3.94%	4.34 ± 1.08
Right hippocampus	MRI U-Net	82.00 ± 3.42%	90.42 ± 2.99%	75.28 ± 5.54%	3.79 ± 0.74
	Cropped MRI U-Net	85.62 ± 1.92%	88.56 ± 2.93%	83.03 ± 3.68%	3.54 ± 0.80
	Shape MRI U-Net	86.06 ± 2.01%	88.20 ± 3.46%	84.21 ± 3.70%	3.47 ± 0.88
	Tissue MRI U-Net	73.59 ± 6.64%	75.42 ± 6.97%	72.03 ± 7.30%	4.19 ± 0.88
	Autocontext MRI U-Net	79.24 ± 6.07%	77.19 ± 6.41%	81.52 ± 6.37%	3.03 ± 0.60
	FreeSurfer 6.0	80.21 ± 3.86%	83.63 ± 4.35%	77.31 ± 5.36%	4.50 ± 1.23

*The proposed pipelines were re-trained on the dataset from the AddNeuroMed cohort and tested on the data from the ADNI cohort, which were previously used for training. The performance of the methods is presented in terms of Dice score, precision, recall, and Hausdorff distance. The performance is reported also for the segmentations obtained using FreeSurfer 6.0 on the same data. All evaluation metrics are expressed as mean ± standard deviation.*

Two major differences could be found compared to the previous cross-cohort evaluation. First, Tissue MRI U-Net showed a much worse performance in terms of Dice score, precision, and recall. Second, all the other deep-learning based methods resulted in having both higher precision and lower recall compared to the previous analysis.

### Testing on a Larger ADNI Dataset

**FIGURE 5:**
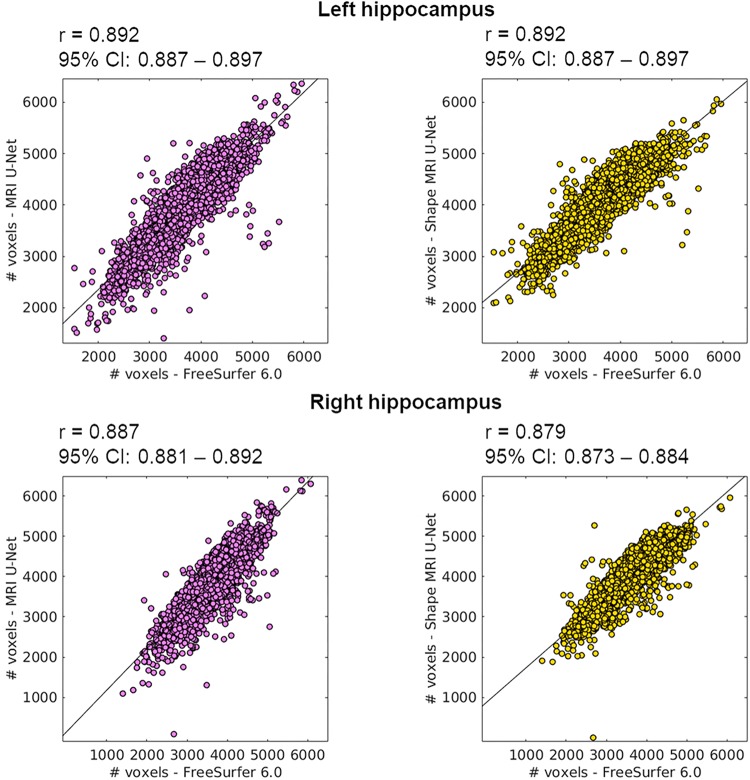
Correlation between the volume of the segmentations obtained using the proposed methods (on the large ADNI test dataset) and those from FreeSurfer 6.0. The Pearson correlation coefficient (*r*) is reported together with its 95% confidence interval (95% CI). Results are reported for both the left (top row) and right (bottom row) hippocampus, and for both MRI U-Net (pink) and Shape MRI U-Net (yellow) in comparison with FreeSurfer 6.0. The volumes are plotted and expressed in terms of number of voxels in the region of interest.

The 5948 additional cases from the ADNI cohort were segmented using the networks trained on the above-described balanced ADNI dataset of 54 subjects. The correlation coefficients of the volumetric results were rather high and consistent between each of the two tested pipelines and FreeSurfer, as can be observed in Figure 5. For the sake of completeness, we also computed the correlation between the two present U-Net based pipelines as well, which resulted in a correlation coefficient of 0.952 for the left and 0.958 for the right hippocampus. Thus, there is a higher correlation between the tested DL-based pipelines than between either of these pipelines and FreeSurfer.

**FIGURE 6:**
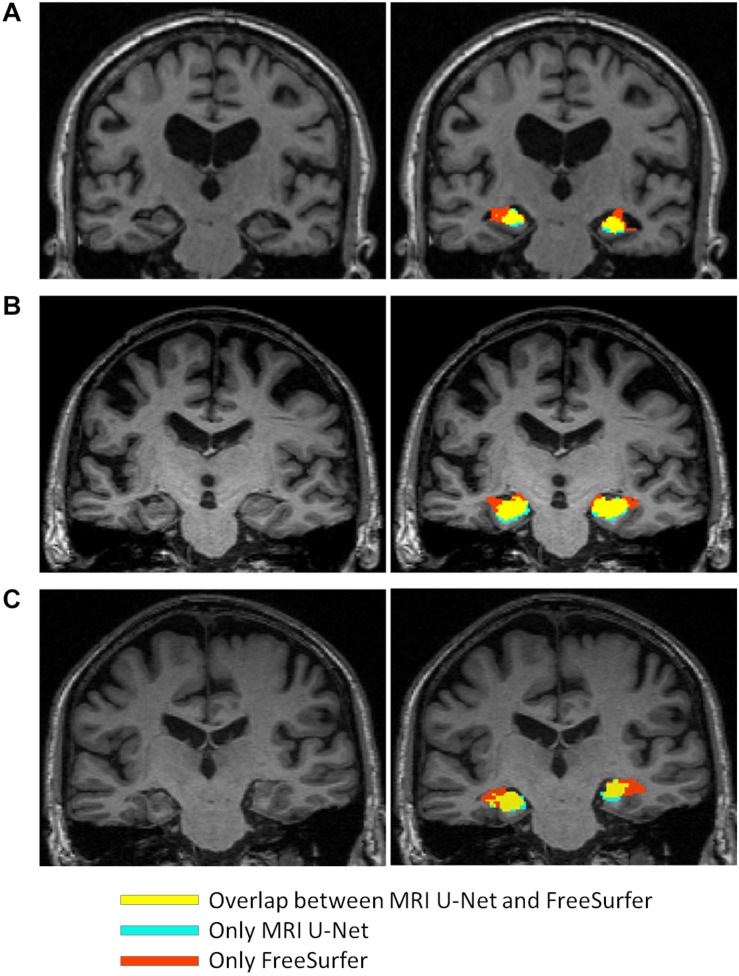
Comparison between the segmentation result obtained from using MRI U-Net and the one from FreeSurfer 6.0 in some examples slices of different subjects from the large ADNI test dataset. The yellow areas indicate the overlap between the two segmentation results, the light blue ones correspond only to the MRI U-Net segmentation, and the red ones only to the FreeSurfer segmentation. All represented subjects belong to the group of 14 cases for which the ratio between the deep-learning based volumes (from all three pipelines) and the volume from the FreeSurfer segmentation had a value that was considered as outlier. In **(A)** and **(B)**, FreeSurfer over-estimates the hippocampal region compared to MRI U-Net that achieves a more reliable estimation. In **(C)**, an unusual shape of the anatomical gyri surrounding the hippocampus is present and both methods result in some segmentation errors, but MRI U-Net achieves a more accurate result compared to the large overestimation obtained by using FreeSurfer.

The scatter plots of Figure 5 highlight the presence of a few outliers, whose number appears to be higher using MRI U-Net but decreases with Shape MRI U-Net. For each of the proposed pipelines, we computed the hippocampal volume of every subject—obtained after applying one of the given segmentation pipelines—divided by the hippocampal volume obtained, instead, from FreeSurfer on the same subject. These ratios were then used to extract a measure of the amount of outliers. We defined as outliers all those subjects that, for a specific segmentation pipeline, showed a volumetric ratio deviating from the median ratio by at least three times the median absolute deviation. The results confirmed what could be seen from the plot. Indeed, for the left and right hippocampus, respectively, 104 and 140 outliers were identified using MRI U-Net, while 84 and 96 using Shape MRI U-Net. Of these subjects, 14 appeared as outliers (for both left and right hippocampus) in all three pipelines. All these 14 cases were either MCI subjects or AD patients and examples of the segmentation results in some of those are shown in Figure 6. An expert was asked to compare the segmentations obtained from FreeSurfer with those from MRI U-Net (which, as in Figure 6, was chosen as reference DL-based segmentation method for this evaluation) in these 14 subjects. In all 14 cases, FreeSurfer showed segmentation errors. With MRI U-Net, instead, three out of these 14 cases showed good segmentation results, five out of 14 showed inaccurate but better results than FreeSurfer, while the remaining six cases were classified as segmentation errors in the same manner as FreeSurfer. Moreover, in Figure 5, the plot for the left hippocampal segmentation using MRI U-Net and both plots for the right hippocampus show one specific point that has a very low volume (in some cases very close to zero). This point corresponds to the same subject in all of these three cases. The original MRI scan of this subject was visually inspected, and it was found to be affected by artifacts that made the identification of the hippocampus particularly challenging. The result obtained on the same subjects on the left hippocampus using Shape MRI U-Net was also inaccurate, even if characterized by a larger amount of voxels.

Two additional similarity metrics (i.e., Dice score and Hausdorff distance) have been computed to compare the results from FreeSurfer with those from both MRI U-Net and Shape MRI U-Net (see Supplementary Table S4). These results showed a rather high consistency between these methods, with an average Dice score close to 79% for the comparison with MRI U-Net, and around 82% for Shape MRI U-Net. Also the Hausdorff distance was rather low (i.e., around 4 voxels in average) for all methods.

We also investigated whether there is a statistically significant difference in the normalized hippocampal volume between the three diagnostic groups of interest, i.e., AD, MCI, and HC. All the three analyzed segmentation methods (MRI U-Net, Shape MRI U-Net, and FreeSurfer 6.0) resulted in statistically significant differences between all three diagnostic groups. As can be seen in Table 5, the lowest normalized hippocampal volumes were always found in the AD patients, and the highest in the HCs. We then investigated the diagnostic prediction power by computing the AUC, sensitivity, and specificity of three logistic regression models that were fitted to classify AD vs. HC, AD vs. MCI, and MCI vs. HC by using the above-mentioned normalized measurements. The results, which are reported in Table 6, show that, for all three segmentations methods, a rather good prediction power is achieved when comparing AD subjects and HC, with a AUC that is above 0.80. Instead, the task of distinguishing AD from MCI and MCI from HC subjects is more challenging, with an AUC of 0.68 for all three methods in the classification of AD vs. MCI and slightly lower AUCs for the classification of MCI vs. HC. Sensitivity and specificity measurements are also shown to be consistent with the AUC across methods and classification tasks. Moreover, the DL-based methods have also shown to have a slightly higher performance compared to FreeSurfer, given their overall higher evaluation metrics.

**TABLE 5:** Volumetric differences in the hippocampal volume between diagnostic groups in a subset of subjects from the large ADNI test dataset.

**Region of interest**	**Segmentation method**	**AD (*n* = 93)**	**MCI (*n* = 267)**	**HC (*n* = 154)**	***p*-value (one-way ANOVA)**
Left hippocampus	MRI U-Net	3.54 ± 0.66 cm^3^	3.95 ± 0.60 cm^3^	4.30 ± 0.54 cm^3^	*p* < 0.001
	Shape MRI U-Net	3.67 ± 0.60 cm^3^	4.04 ± 0.59 cm^3^	4.39 ± 0.51 cm^3^	*p* < 0.001
	FreeSurfer 6.0	3.25 ± 0.60 cm^3^	3.60 ± 0.63 cm^3^	3.99 ± 0.58 cm^3^	*p* < 0.001
Right hippocampus	MRI U-Net	3.38 ± 0.66 cm^3^	3.81 ± 0.66 cm^3^	4.25 ± 0.54 cm^3^	*p* < 0.001
	Shape MRI U-Net	3.56 ± 0.61 cm^3^	3.93 ± 0.62 cm^3^	4.34 ± 0.50 cm^3^	*p* < 0.001
	FreeSurfer 6.0	3.16 ± 0.59 cm^3^	3.50 ± 0.63 cm^3^	3.89 ± 0.54 cm^3^	*p* < 0.001

*For each subject, the hippocampal volume was multiplied by the ratio between the average ICV and the specific subject’s ICV. Results are reported for baseline measurements as mean ± standard deviation for each of the three diagnostic groups of interest, i.e., AD patients, MCI subjects, and healthy controls. Only subjects whose diagnosis did not change within 2 years after the first measurement were selected. The number of subject *n* in each group is indicated in brackets. For each method, a one-way ANOVA test was conducted for comparing the three diagnostic groups.*

**TABLE 6:** Prediction power of using the normalized hippocampal volume measurements to classify AD vs. HC, AD vs. MCI, and MCI vs. HC.

**Segmentation method**	**AD vs. HC**	**AD vs. MCI**	**MCI vs. HC**
MRI U-Net	AUC = 0.85	AUC = 0.68	AUC = 0.67
	Sensitivity = 0.75	Sensitivity = 0.60	Sensitivity = 0.62
	Specificity = 0.82	Specificity = 0.65	Specificity = 0.69
Shape MRI U-Net	AUC = 0.84	AUC = 0.68	AUC = 0.65
	Sensitivity = 0.73	Sensitivity = 0.65	Sensitivity = 0.59
	Specificity = 0.80	Specificity = 0.60	Specificity = 0.66
FreeSurfer 6.0	AUC = 0.82	AUC = 0.68	AUC = 0.64
	Sensitivity = 0.73	Sensitivity = 0.66	Sensitivity = 0.60
	Specificity = 0.73	Specificity = 0.60	Specificity = 0.62

*The diagnostic prediction power was analyzed by fitting three different logistic regression model (one for each binary classification case) and computing its AUC, sensitivity, and specificity. The model was fitted to give the probability of a subject to belong to a certain diagnostic group as a function of the hippocampal volume multiplied by the ratio between the average ICV and the specific subject’s ICV. Sensitivity and specificity were computed at a threshold of 0.5.*

Finally, in this dataset, we computed the CCCs between the hippocampal volumes from all the available pairs of subsequent test–retest scans from the same subject at the same time point. For the left and right hippocampus, respectively, the CCC resulted in 0.988 and 0.977 with Shape MRI U-Net, 0.989 and 0.986 with MRI U-Net, and in 0.969 and 0.963 with FreeSurfer 6.0.

## Discussion

### Comparison Between the Implemented Pipelines

In this work, three different U-Net based segmentation pipelines were proposed: MRI U-Net, Cropped MRI U-Net, and Shape MRI U-Net. All three methods were shown to be accurate and quick tools for the automatic segmentation of the hippocampus from brain MRI data.

#### Single-Cohort Analysis

The first presented method, MRI U-Net, constitutes the simplest architecture, which takes the original MRI image as input and performs the segmentation using three orthogonal U-Nets. When testing its performance through cross validation on 54 subjects from the ADNI dataset, it was shown to achieve an excellent accuracy (average Dice score of approximately 90%) which was equal to each of the other two proposed and more elaborate methods (Cropped MRI U-Net and Shape MRI U-Net). It also yielded higher accuracy than the software FreeSurfer. To some extent, this was expected since the segmentation protocol of the ground-truth masks coincides with that used to train the network, while it inevitably differs from the atlas on which the FreeSurfer segmentation is based (Fischl et al., 2002). However, given the very high difference in performance between the two methods (i.e., around 10% of improvement in the Dice score), we believe that such comparison is valuable and worth being reported in order to give a measure of how DL-based methods are outperforming older—but still widely used and established—brain image processing software.

These results suggest that, when the training and test set come from the same cohort, the use of the simple T1-weighted scan as input image is more efficient than both using just a portion of the scan (cropped around the hippocampus) and including context information. The step of cropping the image around the hippocampus is probably not needed for the network to increase its performance because data from the same cohort have the same size and very similar scanning quality, and therefore the localization and size of the hippocampal region is quite consistent across images. As regards the lack of improvement by adding shape context layers, it is probably due to the fact that a high accuracy can already be reached by using the preliminary single-channel networks and, as already observed in a previous study (Wang and Smedby, 2017), the inclusion of shape information is most valuable when the structure to be segmented is rather challenging.

The analysis of each independent U-Net (i.e., trained for each view separately) was also useful to better understand the differences between the three approaches, which, globally, seem to be very similar to each other. The coronal view is typically the most used view to perform manual hippocampal segmentation. However, its morphological details are not always sufficient to achieve an accurate results, so the axial and sagittal views have to be checked as well (Boccardi et al., 2015a). Therefore, a superior performance on the coronal view was expected on all the trained U-Nets. However, in MRI U-Net, the best performing network was shown to be the one trained on axial slices, suggesting that this model is able to capture some important image features that differ from those used by the human raters. On the other hand, both Cropped MRI U-Net and Shape MRI U-Net showed a slightly superior performance on the coronal view, which is more consistent with what happens in practice when the segmentation is performed by expert radiologists. Moreover, Cropped MRI U-Net resulted in a relevantly low performance on the sagittal view compared to all other views. In particular, the high average Hausdorff distance suggests the presence of several geometric errors, which are then corrected by integrating the information from the other two views. This could not be observed on Shape MRI U-Net, suggesting that the use of shape information on the sagittal view can help to prevent the occurrence of such geometric errors.

#### Cross-Cohort Analysis

When the networks trained on the ADNI cohort were tested on a dataset from the AddNeuroMed cohort, the observed differences between the three implemented architectures were subject to a consistent change.

In terms of overall accuracy, MRI U-Net and FreeSurfer, which both process the data by receiving as input only the original T1-weighted image, showed a very similar performance. The main difference between them is a lower precision and higher recall obtained, in average, by using MRI U-Net. The lower precision may be due to an over-estimation of the hippocampal mask in regions where hippocampal atrophy is present. This suggests the difficulties of training a network with enough atrophic patterns to be able to obtain accurate segmentations also on new unseen data. On the other hand, the under-estimations obtained by FreeSurfer may be related to other types of segmentation errors in atrophic hippocampal areas as well, as suggested also by the general decrease in performance in MCI and AD subjects (Figure 4). This issue will be subject to future investigations.

Furthermore, a clearly higher accuracy was now observed by employing Cropped MRI U-Net and, even more, Shape MRI U-Net. Therefore, when segmenting new unseen data that differ from those used during training (for example, in terms of image size, scanner types, and image quality), it seems to be motivated to perform a further processing step adding information to the simple MRI scan. A big improvement in the accuracy was seen already by simply cropping the image around the center of gravity of the preliminary hippocampal segmentation, suggesting that already this step largely harmonizes the input images to those used during training. This could be explained by the fact that the second U-net is dealing with a much smaller field of view therefore is less likely to be disturbed by imaging or structure changes outside the core region. On the other hand, compared to Cropped MRI U-Net, the inclusion of shape context layers was also shown to lead to slight, but yet statistically significant, improvements in terms of Dice score and precision. This result supports what has already been observed in the previous section: when a high accuracy in the segmentation is achieved from the simple MRI U-Net implementation, adding shape information does not improve the result; on the other hand, when the MRI U-Net segmentation is more challenging (for example, in this case, due to discrepancies between training and test data), the shape context layers—together with the cropping step—can help to increase the accuracy.

The computation of the evaluation metrics for each independent 2D U-Net also allowed to highlight certain differences between the three approaches that cannot be captured from their global performance. In general, by analyzing the performance of each view independently, the advantage of using Cropped MRI U-Net over MRI U-Net is less noticeable, given its larger differences between views in terms of accuracy, as well as generally higher Hausdorff distance. However, as described above, merging the information from all views together seem to stabilize the result and discard many of the FP that affect the simple 2D-based results. This highlights the importance of integrating information from all views together in order to obtain more reliable segmentation results. This is very consistent with what is suggested for the manual HarP segmentation protocol, i.e., the segmentation must be performed using all views together in order to achieve accurate results. Moreover, similarly to what was observed for the single-cohort analysis, the inclusion of shape context appears again useful to improve the performance not only globally in 3D, but also on a 2D basis. Its performance on all views is indeed superior to the one of both Cropped MRI U-Net and of MRI U-Nets.

The positive contribution of adding the step of shape model fitting is further supported by the comparison with two other types of context information. Indeed, Shape MRI U-Net was found to be the most successful method among all those tested. The difference between Shape MRI U-Net and all other approaches was indeed shown to be statistically significant for most of the evaluation metrics. In particular, as regards Autocontext MRI U-Net, we believe that the network tends to learn mainly from the first U-Net-based segmentation without extracting much more information from the T1 volume. This would explain why there is no real improvement in performance and its accuracy is rather similar to that of MRI U-Net. In the case of Tissue MRI U-Net, instead, we think that automatic tissue types segmentations may tend to fail in some locations. Therefore, this would provide “misleading” information as input to the network, which makes this approach not robust.

The above-discussed observations could be made also when training and test set were switched. Indeed, the average evaluation metrics were quite consistent to those of the first cross-cohort analysis and, also in this case, Shape MRI U-Net showed, overall, the best performance. Only two main differences could be found compared to the previous analysis. First of all, the accuracy of Tissue MRI U-Net got much worse. This could be justified by the fact that, in the AddNeuroMed dataset, the image quality is generally lower, also because of the field strength that is limited to 1.5 T in all subject. This may lead to more imprecise tissue type segmentations used during training, which cause a further degrading of the performance during the test phase on a new dataset. Moreover, for all the other DL-based pipelines, the precision and the recall were, respectively, higher and lower compared to the previous analysis. This result was expected because the training and test sets have been simply switched and therefore possible over-estimations in the first cross-cohort evaluation are likely to result in under-estimations in the second one.

Finally, it should be noted that, despite the increase in performance with Shape MRI U-Net on both cross-cohort analyses, the segmentation accuracy is still lower than the one obtained using cross-validation on the dataset from the ADNI cohort (presented in section “Single-Cohort Evaluation”). However, this was expected due to both the above-discussed discrepancy between training and test cohort, as well as the inter-rater differences when generating the ground-truth segmentations. The experience of the rater (in terms of familiarity with the segmentation task itself, the given image quality and the specific MRI protocol) can indeed affect the manual delineation of the segmentation masks.

#### Analysis on the Larger ADNI Dataset

For the last and largest dataset, where ground-truth masks were not available and visually checking the accuracy was not feasible due to the large amount of data, the performance of the networks was checked by comparing the hippocampal volumes with those obtained using FreeSurfer. This approach clearly has limitations, since it cannot give a detailed measure of the accuracy of the method in this new dataset and could not reveal relevant differences between the three proposed methods. However, the high correlation coefficients (presented in Figure 5) and similarity metrics (Supplementary Table S4) between the proposed methods and FreeSurfer suggest both a valid and consistent performance for all the present methods.

From Figure 5, it is possible to observe that FreeSurfer tends to provide, in general, smaller segmentations compared to the DL-based methods. This is in agreement with what was discussed in Section “Cross-Cohort Analysis” when analyzing the performance on the AddNeuroMed dataset. Indeed, in this case, FreeSurfer was shown to have higher average precision and lower average recall.

Furthermore, the number of identified outliers was low in comparison with the size of the dataset, which further supports the consistency of the results across subjects. On the other hand, the visual inspection of some subjects identified as outliers actually revealed a segmentation result from MRI U-Net that did not appear to be less accurate than the one obtained from FreeSurfer, as shown in Figure 6. This fact further exposes the limitations of not having a ground-truth mask to validate the performance. On the other hand, it also suggests that the results of the proposed DL-based approaches are promising in comparison with other established methods, especially when dealing with potential clinical cases (since no outliers belonged to the HC group).

When comparing FreeSurfer with the two proposed DL-based methods in terms of Dice score and Hausdorff distance, a rather high consistency could also be observed, especially for Shape MRI U-Net that showed, in average, a higher Dice score. The resulting metrics are also rather consistent with the results obtained when analyzing the performance of FreeSurfer both in the single-cohort and the cross-cohort analyses. This was expected because, as opposed to FreeSurfer, the present U-Net-based methods were all trained on the HarP protocol used for the manual segmentations too.

The availability of pairs of scans acquired from the same subjects at the same time point also allowed us to perform a test–retest analysis. This resulted in a very high CCC (i.e., between 0.977 and 0.989) in the hippocampal volumes between two subsequent scans with both the tested methods, i.e., MRI U-Net and Shape MRI U-Net. While the results obtained in the above-described single- and cross-cohort analyses show the accuracy of the method, these high coefficients in the test–retest investigation demonstrate the reproducibility of the proposed techniques. Moreover, FreeSurfer also resulted in slightly lower CCCs (between 0.963 and 0.969), showing how the present methods are to some extent more reproducible.

### Comparison Between Diagnostic Groups

The performance of the three proposed methods was proven to be satisfactory in all three analyzed diagnostic groups: HC, MCI, and AD patients. By computing the Dice score separately for each group, we found that the segmentation accuracy is quite consistent across the groups.

When the network was initially tested through cross-validation on the dataset of 54 subjects from the ADNI cohort, the AD patients’ group was the only one showing a lower performance with respect to the other two. However, this difference was rather small, approximately 1%. The difference between subject groups slightly increased when testing the network on the dataset from the AddNeuroMed cohort, which was different from the cohort of the training data. Indeed, in this case, a little loss in performance was seen already in the MCI subjects, for which the Dice score showed an average decrease of between 1 and 3% compared to HC. In AD patients, the average decrease was between 2 and 4% relative to HC. Observing a slightly better accuracy in the images from HCs was expected. Indeed, MCI subjects and, even more, AD patients are expected to present patterns of hippocampal atrophy, which is strongly related to the severity stage of the disease. These patterns are likely to be quite heterogeneous if compared to the typical hippocampal structure that can be observed on a healthy brain, making the learning of the network more challenging for such diagnostic cases.

Despite the little loss in performance on AD patients, the accuracy of the proposed methods was more satisfactory than the one obtained by applying the automatic segmentation pipeline from FreeSurfer 6.0. FreeSurfer is also affected by a loss in accuracy when segmenting AD patients compared to HC and the magnitude of such loss was always higher than the ones obtained from the presented DL pipelines. These results suggest that the choice of a U-Net-based approach could also be favorable when good segmentation accuracy is needed on brain images from dementia patients. This aspect is particularly important for a medical segmentation tool to be potentially used both in a clinical and a research setting. Indeed, the more accurate the segmentation is, the more reliable the estimations of hippocampal volume and shape will be. Such geometrical features have been shown to be strongly related to the disease progression, and therefore it is crucial to achieve an accurate segmentation also on demented subjects and not only on healthy ones.

The potential of the proposed methods to be used in a clinical framework was also further shown by the comparison between the normalized hippocampal volumes of the three diagnostic groups present in the large ADNI dataset. All present methodologies show significant differences in the distribution of the hippocampal volumes between groups. In particular, the lowest average volume was found in the AD subjects and the highest in the HCs. This suggests that the present methods can capture the differences in volume caused by the atrophy that is typical of the disease progression.

The usefulness of these volumetric differences between groups was further investigated by fitting logistic regression models to predict the diagnosis of a subject. DL-based methods showed a better performance than FreeSurfer 6.0 and the highest AUC (always above 0.80) could be achieved in the classification of AD vs. HC. Similar diagnosis classification tasks have already been investigated in previous literature leading to similar results. Indeed, in a study by Westman et al. (2011b), manual hippocampal segmentations were employed to define multivariate analysis models for diagnosis prediction, obtaining a sensitivity and specificity of, respectively, 87 and 90% for the AD vs. HC classification. Instead, for AD vs. MCI and MCI vs. HC, those evaluation metrics dropped to approximately 70% in all cases. In a later study by Voevodskaya et al. (2014), FreeSurfer 5.1 was used to extract normalized hippocampal volumes from ADNI data and the AUC was computed for three different linear regression models fitted for the same classification tasks. Also in this case, the best result was obtained with AD vs. HC with an AUC of 0.90, while poorer performance was achieved with the other two models. Therefore, our results reflect what has already been observed in literature, i.e., the potential of using accurate hippocampal segmentation methods to improve the diagnosis of AD and its discrimination from healthy cases. Even though there are differences between different studies in their values of AUC, sensitivity, and specificity, it has to be noted that such discrepancies can be due to different factors. First, the number of analyzed subjects and the model definition can highly influence the results, e.g., the model could be affected by overfitting. Moreover, the type and accuracy of the segmentation method being used can also affect the performance. In addition, the patterns of brain atrophy in AD are heterogeneous and it has been estimated that approximately 23% of AD patients are minimally affected by hippocampal atrophy (Poulakis et al., 2018). Therefore, the presence of this type of patients in the dataset can also affect the prediction power of a model based only on hippocampal volume. However, in general, our study is particularly consistent with the others in terms of the difference in performance between the AD vs. HC classification compared to the other two classification tasks. This discrepancy between classifiers, though, will always be expected given the typical patterns of disease progression, since the differences in atrophy between AD and MCI subjects, as well as between MCI and HC, are inevitably smaller compared to the differences between AD patients and healthy subjects.

### Computational Time

The present pipelines were proven to be successful not only in terms of segmentation accuracy, but also in terms of computational speed, which varied between approximately 30 and 150 s depending on the architecture being used. Time efficiency is another important aspect to be taken into account in order to use a segmentation tool in a clinical framework as an aid for performing a diagnosis. Therefore, a DL-based solution is promising in the context of potential clinical use. However, it has to be noted that a computationally slower software as FreeSurfer provides, together with the hippocampus, the segmentation masks for many other gray and white matter structures, as opposed to the present study that is focused only on hippocampal segmentation. This implies that the choice of the most efficient segmentation method is strongly dependent on the application of interest and on the level of accuracy that is required from the segmentation result.

### Limitations and Future Work

The present work investigates the use of a DL architecture for an image segmentation task that is of particular interest for AD research. Indeed, achieving an accurate hippocampal segmentation is a crucial task for aiding research in the early diagnosis of the disorder. Moreover, precise standards on how to perform a good manual segmentation of the hippocampus are available, making it easier to obtain ground-truth masks to train the network with. However, the number of training data used for the present work was still quite limited. This issue was approached by using data augmentation, but in the future we plan to expand the training dataset by adding more manual segmentations performed by experts. Moreover, it would be useful to obtain manual segmentations of other brain regions, whose geometrical information could be integrated with those from the hippocampus. Therefore, we also aim at extending the study by testing the proposed pipelines on other brain structures that are both of interest for Alzheimer’s research and known to be particularly challenging for segmentation, such as the entorhinal cortex. In particular, we want to investigate whether the inclusion of shape information can be even more useful in such a context.

In addition, we would like to change our architecture by using 3D U-Nets instead of the three independent 2D U-Nets. In the present work, an implementation using 2D U-Nets was employed mainly because of the limited 3D training data samples and its advantage over a 3D implementation in terms of memory usage. However, in the future, we would like to test whether the direct use of 3D information could further improve the segmentation accuracy in any of the proposed pipelines.

Moreover, one of the limitations of this study is that the inclusion of shape information encoded in statistical shape models is not entirely new, as already presented in a previous study by Wang and Smedby (2017). In the future, we aim at investigating a wider range of shape descriptors that could possibly further improve the performance of our shape-aware segmentation pipeline. However, besides the different field of application (hippocampal segmentation instead of heart segmentation), the main contribution of this study compared to the one by Wang and Smedby (2017) is the extensive analysis of the performance of Shape MRI U-Net on larger datasets of subjects from different diagnostic groups and cohorts, as well as the comparison with two other types of context-aware architectures. The present work provides a new insight on how the inclusion of *a priori* shape information can be employed in cross-cohort analyses or, more in general, when a testing dataset was not used at the time of training. In the context of hippocampal segmentation, the use of shape information was shown to be indeed more successful than other types of *a priori* information that could be extracted from the given anatomical structures. The integration and comparison with other *a priori* information, as well as the analyses of new cohorts, could be investigated in the future to further confirm the present findings.

Finally, in this study, the MRI scans underwent only a couple of preprocessing stages, i.e., resampling and intensity normalization. A further harmonization of the inputs was later obtained by cropping the images on Cropped MRI U-Net, as well as including the normalized shape models on Shape MRI U-Net. This choice was made to keep the pipeline as simple and quick as possible. However, we would like to investigate whether the addition of a few other preprocessing steps, such as skull stripping, could help improving the performance of MRI U-Net.

## Conclusion

The present work has proposed an accurate and fast method for automatic segmentation of the hippocampus using U-Net-based DNNs together with statistical shape modeling.

A simpler and quicker U-Net architecture, which simply uses the original MRI scan as input image, achieved already excellent results in a first single-cohort analysis. However, the proposed implementation using shape context was shown to be more successful with data from a new unseen cohort by significantly improving the segmentation accuracy. These results suggest that the inclusion of shape information may make the method more robust in cases where the segmentation task is more challenging.

Our promising results across different diagnostic groups suggest that the proposed method could not only be used as a possible substitute for other existing segmentation tools, but may also have a potential as an aid for studying and diagnosing neurodegenerative disorders.

## Data Availability Statement

Publicly available datasets were analyzed in this study. These data can be found here: http://adni.loni.usc.edu/.

## Ethics Statement

The data acquisition for the AddNeuroMed study was approved by the Regional Ethics Board Stockholm 2013/694-31. Data were acquired after written informed consent.

## Author Contributions

CW, ÖS, EW, and IB contributed to the conception and design of the study. OL provided the manual segmentations for the AddNeuroMed dataset. J-SM organized the database and provided the FreeSurfer segmentations. IB and CW developed the segmentation pipeline and shape model. IB, CW, ÖS, EW, and OL worked on the final method evaluation and analysis of the results. All authors contributed to the writing of the manuscript and its revision, as well as approved the submitted version.

## Conflict of Interest

The authors declare that the research was conducted in the absence of any commercial or financial relationships that could be construed as a potential conflict of interest.

## Footnotes

1 adni.loni.usc.edu

2 https://keras.io

3 http://surfer.nmr.mgh.harvard.edu/
