# Supplementary material for: Shape Information Improves the Cross-Cohort Performance of Deep Learning-Based Segmentation of the Hippocampus
Source: Front Neurosci. 2020 Jan 24;14:15. doi: 10.3389/fnins.2020.00015 (PMC7081773; doi:10.3389/fnins.2020.00015)
Supplement: Supplementary file 1 [file Table_1.DOCX]

Supplementary Table 1. Single-cohort evaluation. The performance of the proposed methods (in terms of Dice score, precision, recall and Hausdorff distance) was computed through nine-fold cross validation and separately for each view. All evaluation metrics are expressed as mean ± standard deviation.

| **Region of interest** | **Segmentation method** | **Dice score** | **Precision** | **Recall** | **Hausdorff distance (in voxels)** |
| --- | --- | --- | --- | --- | --- |
| Left hippocampus | MRI U-Net | Axial:  90.78% ± 1.43%  Coronal:  89.56% ± 3.27%  Sagittal:  88.40% ± 4.11% | Axial:  90.26% ± 1.87%  Coronal:  89.95% ± 1.61%  Sagittal:  91.16% ± 1.68% | Axial:  91.36% ± 2.25%  Coronal:  92.57% ± 2.16%  Sagittal:  90.76% ± 2.17% | Axial:  2.19 ± 0.83  Coronal:  2.09 ± 0.67  Sagittal:  2.19 ± 0.49 |
|  | Cropped  MRI U-Net | Axial:  89.15% ± 1.35%  Coronal:  90.16% ± 1.17%  Sagittal:  83.15% ± 4.89% | Axial:  86.80% ± 2.02%  Coronal:  87.56% ± 1.73%  Sagittal:  76.56% ± 7.55% | Axial:  91.67% ± 2.00%  Coronal:  92.95% ± 1.76%  Sagittal:  91.42% ± 2.06% | Axial:  3.88 ± 7.02  Coronal:  2.66 ± 3.86  Sagittal:  33.98 ± 4.94 |
|  | Shape  MRI U-Net | Axial:  89.13% ± 1.39%  Coronal:  90.15% ± 1.40%  Sagittal:  89.76% ± 1.48% | Axial:  87.93% ± 2.40%  Coronal:  88.03% ± 2.12%  Sagittal:  87.39% ± 2.30% | Axial:  90.44% ± 2.05%  Coronal:  92.43% ± 1.84%  Sagittal:  92.31% ± 1.66% | Axial:  2.20 ± 0.47  Coronal:  2.27 ± 0.60  Sagittal:  2.25 ± 0.74 |
| Right hippocampus | MRI U-Net | Axial:  90.86% ± 1.33%  Coronal:  89.11% ± 3.48%  Sagittal:  87.74% ± 3.02% | Axial:  90.42% ± 1.76%  Coronal:  89.96% ± 1.69%  Sagittal:  90.85% ± 1.97% | Axial:  91.36% ± 1.90%  Coronal:  93.10% ± 1.64%  Sagittal:  90.69% ± 2.19% | Axial:  2.35 ± 0.59  Coronal:  2.27 ± 0.75  Sagittal:  2.44 ± 0.72 |
|  | Cropped  MRI U-Net | Axial:  89.17% ± 1.27%  Coronal:  90.10% ± 1.05%  Sagittal:  83.91% ± 4.86% | Axial:  86.81% ± 2.38%  Coronal:  87.40% ± 2.30%  Sagittal:  77.94% ± 7.57% | Axial:  91.74% ± 2.11%  Coronal:  93.05% ± 1.83%  Sagittal:  91.32% ± 2.08% | Axial:  2.40 ± 0.70  Coronal:  2.90 ± 3.15  Sagittal:  32.71 ± 4.88 |
|  | Shape  MRI U-Net | Axial:  89.39% ± 1.37%  Coronal:  90.10% ± 1.30%  Sagittal:  89.88% ± 1.38% | Axial:  88.43% ± 2.88%  Coronal:  88.06% ± 2.62%  Sagittal:  87.68% ± 2.74% | Axial:  90.49% ± 2.23%  Coronal:  92.34% ± 2.02%  Sagittal:  92.28% ± 1.99% | Axial:  2.29 ± 0.51  Coronal:  2.42 ± 0.93  Sagittal:  2.49 ± 0.99 |
