# Supplementary material for: Shape Information Improves the Cross-Cohort Performance of Deep Learning-Based Segmentation of the Hippocampus
Source: Front Neurosci. 2020 Jan 24;14:15. doi: 10.3389/fnins.2020.00015 (PMC7081773; doi:10.3389/fnins.2020.00015)
Supplement: Supplementary file 2 [file Table_2.DOCX]

**Supplementary Table 2.** Cross-cohort evaluation. The proposed methods were tested on a new unseen dataset from a different cohort (i.e. AddNeuroMed cohort) than the one used for training. The performance (in terms of Dice score, precision, recall and Hausdorff distance) is reported separately for each view. All evaluation metrics are expressed as mean ± standard deviation.

| **Region of interest** | **Segmentation method** | **Dice score** | **Precision** | **Recall** | **Hausdorff distance (in voxels)** |
| --- | --- | --- | --- | --- | --- |
| Left hippocampus | MRI U-Net | Axial:  81.92% ± 2.43%  Coronal:  80.29% ± 5.55%  Sagittal:  80.70% ± 4.21% | Axial:  77.90% ± 4.06%  Coronal:  77.10% ± 4.81%  Sagittal:  78.92% ± 4.12% | Axial:  86.63% ± 3.83%  Coronal:  86.86% ± 5.54%  Sagittal:  86.08% ± 3.47% | Axial:  3.48 ± 0.78  Coronal:  3.70 ± 0.99  Sagittal:  3.64 ± 0.85 |
|  | Cropped  MRI U-Net | Axial:  83.74% ± 2.33%  Coronal:  83.31% ± 3.26%  Sagittal:  78.23% ± 4.94% | Axial:  78.11% ± 4.10%  Coronal:  77.33% ± 5.01%  Sagittal:  69.62% ± 8.11% | Axial:  90.45% ± 2.42%  Coronal:  90.64% ± 4.24%  Sagittal:  90.14% ± 2.54% | Axial:  6.75 ± 8.98  Coronal:  5.40 ± 6.67  Sagittal:  30.74 ± 8.42 |
|  | Shape  MRI U-Net | Axial:  83.52% ± 2.43%  Coronal:  84.05% ± 2.71%  Sagittal:  83.57% ± 2.70% | Axial:  78.65% ± 4.76%  Coronal:  78.00% ± 5.13%  Sagittal:  77.28% ± 5.07% | Axial:  89.36% ± 3.50%  Coronal:  91.47% ± 2.98%  Sagittal:  91.35% ± 3.28% | Axial:  3.30 ± 0.91  Coronal:  3.41 ± 0.99  Sagittal:  3.46 ± 0.81 |
| Right hippocampus | MRI U-Net | Axial:  83.17% ± 2.06%  Coronal:  80.47% ± 4.66%  Sagittal:  80.46% ± 4.80% | Axial:  78.18% ± 3.27%  Coronal:  77.37% ± 3.42%  Sagittal:  78.01% ± 3.48% | Axial:  88.97% ± 2.63%  Coronal:  89.25% ± 4.17%  Sagittal:  86.37% ± 4.01% | Axial:  3.77 ± 1.36  Coronal:  3.70 ± 1.08  Sagittal:  3.86 ± 1.09 |
|  | Cropped  MRI U-Net | Axial:  82.46% ± 2.41%  Coronal:  82.37% ± 2.90%  Sagittal:  77.67% ± 4.87% | Axial:  75.77% ± 3.80%  Coronal:  75.52% ± 4.23%  Sagittal:  68.83% ± 7.28% | Axial:  90.61% ± 2.40%  Coronal:  90.78% ± 3.08%  Sagittal:  89.71% ± 2.58% | Axial:  4.88 ± 5.49  Coronal:  3.82 ± 0.95  Sagittal:  28.77 ± 9.16 |
|  | Shape  MRI U-Net | Axial:  82.52% ± 2.55%  Coronal:  83.18% ± 2.93%  Sagittal:  82.39% ± 2.70% | Axial:  76.69% ± 4.50%  Coronal:  76.52% ± 4.66%  Sagittal:  75.59% ± 4.74% | Axial:  89.62% ± 3.35%  Coronal:  91.41% ± 3.46%  Sagittal:  90.84% ± 2.94% | Axial:  3.74 ± 1.19  Coronal:  3.79 ± 1.10  Sagittal:  3.89 ± 1.08 |
