# Supplementary material for: Shape Information Improves the Cross-Cohort Performance of Deep Learning-Based Segmentation of the Hippocampus
Source: Front Neurosci. 2020 Jan 24;14:15. doi: 10.3389/fnins.2020.00015 (PMC7081773; doi:10.3389/fnins.2020.00015)
Supplement: Supplementary file 3 [file Table_3.DOCX]

**Supplementary Table 3.** Pairwise comparisons of the performance of the segmentation methods on the AddNeuroMed test dataset. p-values (with Bonferroni correction) obtained by applying a mixed-effect analysis-of-variance model for each evaluation metric to the data reported in Table 3. Statistically significant results (p<0.05) are shown in **bold**.

| **Dice score** | MRI  U-Net | Cropped  MRI U-Net | Shape  MRI U-Net | Tissue MRI U-Net | Autocontext MRI U-Net | FreeSurfer 6.0 | **Precision** | MRI  U-Net | Cropped  MRI U-Net | Shape  MRI U-Net | Tissue MRI U-Net | Autocontext MRI U-Net | FreeSurfer 6.0 |
| --- | --- | --- | --- | --- | --- | --- | --- | --- | --- | --- | --- | --- | --- |
| MRI U-Net |  | **< 0.001** | **< 0.001** | **< 0.001** | **< 0.001** | 0.607 | MRI U-Net |  | **< 0.001** | **< 0.001** | **< 0.001** | **0.004** | **< 0.001** |
| Cropped MRI U-Net |  |  | **< 0.001** | 0.860 | **< 0.001** | **< 0.001** | Cropped MRI U-Net |  |  | **< 0.001** | 0.055 | **< 0.001** | **0.004** |
| Shape MRI U-Net |  |  |  | **< 0.001** | **< 0.001** | **< 0.001** | Shape MRI U-Net |  |  |  | **0.049** | **< 0.001** | 0.295 |
| Tissue MRI U-Net |  |  |  |  | **< 0.001** | **< 0.001** | Tissue MRI U-Net |  |  |  |  | **< 0.001** | 0.355 |
| Autocontext MRI U-Net |  |  |  |  |  | **< 0.001** | Autocontext MRI U-Net |  |  |  |  |  | **< 0.001** |
| FreeSurfer 6.0 |  |  |  |  |  |  | FreeSurfer 6.0 |  |  |  |  |  |  |
| **Recall** | MRI  U-Net | Cropped  MRI U-Net | Shape  MRI U-Net | Tissue MRI U-Net | Autocontext MRI U-Net | FreeSurfer 6.0 | **Hausdorff Distance** | MRI  U-Net | Cropped  MRI U-Net | Shape  MRI U-Net | Tissue MRI U-Net | Autocontext MRI U-Net | FreeSurfer 6.0 |
| MRI U-Net |  | **< 0.001** | **< 0.001** | **< 0.001** | **< 0.001** | **< 0.001** | MRI U-Net |  | 0.137 | **0.022** | 0.162 | 0.536 | **< 0.001** |
| Cropped MRI U-Net |  |  | 0.459 | **0.002** | **< 0.001** | **< 0.001** | Cropped MRI U-Net |  |  | 0.423 | 0.928 | 0.385 | **< 0.001** |
| Shape MRI U-Net |  |  |  | **< 0.001** | **< 0.001** | **< 0.001** | Shape MRI U-Net |  |  |  | 0.372 | 0.095 | **< 0.001** |
| Tissue MRI U-Net |  |  |  |  | **0.002** | **< 0.001** | Tissue MRI U-Net |  |  |  |  | 0.437 | **< 0.001** |
| Autocontext MRI U-Net |  |  |  |  |  | **< 0.001** | Autocontext MRI U-Net |  |  |  |  |  | **< 0.001** |
| FreeSurfer 6.0 |  |  |  |  |  |  | FreeSurfer 6.0 |  |  |  |  |  |  |
