# Supplementary material for: Shape Information Improves the Cross-Cohort Performance of Deep Learning-Based Segmentation of the Hippocampus
Source: Front Neurosci. 2020 Jan 24;14:15. doi: 10.3389/fnins.2020.00015 (PMC7081773; doi:10.3389/fnins.2020.00015)
Supplement: Supplementary file 4 [file Table_4.DOCX]

**Supplementary Table 4.** Measures of similarity between the segmentation outputs from FreeSurfer 6.0 and the two tested deep learning-based methods (MRI U-Net and Shape MRI U-Net) on the large ADNI test dataset. All metrics are expressed as mean ± standard deviation.

| **Region of interest** | **Segmentation method** | **Dice score** | **Hausdorff distance (in voxels)** |
| --- | --- | --- | --- |
| Left hippocampus | MRI U-Net – FreeSurfer 6.0 | 78.78% ± 2.71% | 3.97 ± 1.11 |
|  | Shape MRI U-Net – FreeSurfer 6.0 | 82.57% ± 2.90% | 4.19 ± 1.37 |
| Right hippocampus | MRI U-Net – FreeSurfer 6.0 | 78.69% ± 3.14% | 4.01 ± 1.25 |
|  | Shape MRI U-Net – FreeSurfer 6.0 | 81.84% ± 3.48% | 4.13 ± 1.36 |
